# Draft Genome Sequence of *Pseudomonas fluorescens* Strain ITEM 17298, Associated with Cheese Spoilage

**DOI:** 10.1128/genomeA.01141-17

**Published:** 2017-10-26

**Authors:** Francesca Fanelli, Vania C. Liuzzi, Laura Quintieri, Giuseppina Mulè, Federico Baruzzi, Antonio F. Logrieco, Leonardo Caputo

**Affiliations:** Institute of Sciences of Food Production, National Research Council, Bari, Italy

## Abstract

*Pseudomonas fluorescens* is a genetically and phenotypically heterogeneous species that is often reported as a spoiler of fresh foods, but it has recently been implicated in clinical infection. In this study, we sequenced the genome of *P. fluorescens* strain ITEM 17298, isolated from mozzarella cheese and able to cause several alterations under cold storage.

## GENOME ANNOUNCEMENT

The *Pseudomonas fluorescens* species complex is one of the most diverse groups within the *Pseudomonas* genus, showing enormous phenotypic and genetic heterogeneity. Members of this group can inhabit several environments, such as water (1), soil (2), plant tissues (3), fungi (4), animals (5), and humans (6), and play important ecological functions, including promotion of plant growth, induction of systemic resistance, and biological control (7). In addition, they have been widely described as milk and fresh cheese spoilers (8–10). 

Recently, *P. fluorescens* strain 84095, isolated and identified from high-moisture mozzarella cheese (11), was characterized for its ability to grow under cold storage. It was reported as being responsible for noticeable changes in the quality and length of shelf life of this fresh cheese, including proteolysis and discoloration (12). This strain was maintained under the accession number ITEM 17298 in the microbial ITEM Culture Collection of the Institute of Sciences of Food Production of Bari, Italy (http://server.ispa.cnr.it/ITEM/Collection).

DNA was extracted from a single colony of *P. fluorescens* strain ITEM 17298 and submitted to IGA Technology Services (Udine, Italy) for whole-genome shotgun sequencing using the Illumina HiSeq2500 platform, and approximately 18 Mb of 125-bp paired-end reads (59.69% G+C content) were obtained. A preliminary evaluation of the quality of the raw data was performed with FastQC software. *De novo* assembly was performed using SPAdes version 3.5.0 software within the Galaxy platform (13), obtaining a total of 318 contigs (247 contigs >200 bp). The overall contiguity of the assembly is good, with an *N*_50_ value of 147 kb; the longest assembled fragment was 406 kb in length (determined with QUAST, available at https://sourceforge.net/projects/quast/files), while the total length of the assembly was 6,321,090 bp.

To date, 114 genomes of *P. fluorescens* have been published, but only 4 of these are related to strains described as causing food spoilage. The publication of this genome sequence will allow comparison between ITEM 17298 and other *P. fluorescens* food spoilers and will provide novel data for studying dairy spoilage bacteria.

### Accession number(s).

This whole-genome shotgun project has been deposited at DDBJ/ENA/GenBank under the accession number NPKB00000000. The version described in this paper is the first version, NPKB01000000.
